# Impact of Large Language Model–Based AI Tools on Physician-Patient Communication: Systematic Review and Meta-Analysis

**DOI:** 10.2196/77307

**Published:** 2026-07-31

**Authors:** Sven Richter, Clara Helene Buszello, Markus Prem, Sophia Willkommen, Elida Hasani, Ortrud Uckermann, Tareq A Juratli, Ilker Y Eyüpoglu, Witold H Polanski

**Affiliations:** 1 Department of Neurosurgery, Medical Faculty and University Hospital Carl Gustav Carus, Technische Universität Dresden, Fetscherstrasse 74, Dresden, 01307, Germany, 49 3514582883; 2 Else Kröner Fresenius Center for Digital Health, Medical Faculty and University Hospital Carl Gustav Carus, Technische Universität Dresden, Dresden, Germany

**Keywords:** large language models, physician-patient communication, artificial intelligence in health care, empathy, ChatGPT, LLMs

## Abstract

**Background:**

Recent advances in large language models (LLMs) such as GPT-3/4 have spurred the development of artificial intelligence (AI) chatbots and advisory tools in medicine. These systems are posited to assist or augment physician-patient communication, potentially improving empathy, clarity, and responsiveness. However, their actual impact on communication outcomes remains uncertain.

**Objective:**

This study aimed to systematically review and meta-analyze peer-reviewed studies (2020‐2025) evaluating how LLM-based interventions affect physician-patient communication, including empathy, clarity, trust, and patient understanding.

**Methods:**

Following PRISMA (Preferred Reporting Items for Systematic Reviews and Meta-Analyses) 2020 guidelines, we searched PubMed/MEDLINE, Embase, Scopus, and Web of Science for studies published from 2020 to 2025 examining LLM or chatbot applications in clinical communication contexts. Eligible designs included randomized, observational, cross-sectional, and qualitative studies. Two reviewers (WHP and SR) independently screened titles or abstracts, assessed full texts, and extracted data on study design, population, LLM type, communication measures, and outcomes. We conducted a qualitative synthesis and random-effects meta-analysis, reporting pooled standardized mean differences or odds ratios with 95% CIs.

**Results:**

From 312 records, 10 studies were included, all quantitative and predominantly cross-sectional. Populations ranged from patients with chronic conditions to health care professionals and laypersons. Outcomes assessed included empathy (8 studies), clarity or information quality (6 studies), satisfaction or usefulness (4 studies), and trust perceptions (2 studies). In 6 direct comparisons of AI- versus physician-generated responses, LLMs were rated significantly higher in empathy in 5 studies. One large study found that chatbot replies were judged empathetic in 45.1% of cases versus 4.6% for physician replies (odds ratio approximately 9.8, *P*<.001). Similarly, ChatGPT-4 answers scored higher in empathy on a 5-point scale than human-written responses (mean 4.18 vs 2.70, *P*<.001). One neurology study showed higher empathy scores (Consultation and Relational Empathy Scale +1.38, *P*<.01) for ChatGPT answers. Only 1 study found no significant empathy difference. LLM content was also longer and more information-rich, improving patient-perceived clarity and understanding. On the other hand, GPT-4 simplified pathology reports, increasing patient comprehension scores (7.98 vs 5.23/10, *P*<.001) and reducing consultation time by 70%. However, AI replies were sometimes less concise or less readable for low-literacy patients. In pooled analyses (*k*=4 studies; total evaluations N=2604), LLM assistance showed a large positive effect on empathy (standardized mean difference 1.02, 95% CI 0.44‐1.60; random-effects model). Patient satisfaction results were mixed. No study directly assessed long-term trust.

**Conclusions:**

Current evidence suggests that LLM-based chatbots can enhance physician-patient communication by producing more empathetic, detailed, and understandable responses. These improvements may positively influence patient experience and engagement. However, LLMs may also generate overly lengthy or occasionally inaccurate advice, emphasizing the need for physician oversight. While meta-analytic findings are promising, robust randomized controlled trials, real-world and longitudinal studies are needed to confirm benefits, assess trust outcomes, and define optimal clinical integration strategies.

## Introduction

Effective communication is the cornerstone of the physician-patient relationship, influencing patient satisfaction, adherence, and health outcomes [1]. Key communication attributes such as empathy, clarity in explanations, and trust-building are widely recognized as markers of quality care [2]. However, physicians today face growing communication challenges [3]. The rise of electronic messaging and telemedicine since 2020 has led to surging volumes of patient inquiries, contributing to physician workload and burnout [4]. Under time pressure, clinicians’ responses to patients may become terse or delayed, potentially lacking empathy or detailed clarification [5]. In this context, artificial intelligence (AI) powered by large language models (LLMs) has emerged as a potential solution to assist with or enhance physician-patient communication [6].

LLMs such as GPT-4, Llama, Gemini, or Claude are AI systems trained on vast text corpora capable of understanding queries and generating human-like responses [7]. Since late 2022, when ChatGPT popularized this technology, there has been intense interest in medical applications of LLMs [8,9]. Early studies demonstrated that ChatGPT could answer medical knowledge questions at near-expert level [10], but beyond factual correctness, a critical question is how communicative these answers are. Anecdotal reports suggested that AI chatbots might excel at expressing empathy or “bedside manner.” For instance, an AI assistant can draft a reply to a patient’s message using a friendly and understanding tone, which the clinician could then review [11]. Proponents argue that this could improve responsiveness and empathy in patient communications while saving clinicians time [12]. On the other hand, concerns have been raised about the authenticity of “artificial empathy,” potential misinformation, and how patients perceive advice created by AI.

Given the rapid development of LLM-driven medical chatbots (eg, symptom checkers, mental health coaches, and patient portal assistants), it is imperative to systematically evaluate their impact on communication outcomes. Prior literature has primarily focused on the technical accuracy of AI or the feasibility of use [13], with less attention to interpersonal aspects such as empathy, trust, and patient-centered communication. This review addresses that gap by examining all recent studies that measured communication-related outcomes of LLM interventions in health care. We summarize evidence on whether and how these AI tools influence empathy, clarity of information exchange, patient satisfaction, and perceived communication quality in physician-patient interactions.

Our objectives were to (1) identify all peer-reviewed studies from 2020 onward that evaluate LLM or chatbot effects on any physician-patient communication dimension, (2) extract and compare the communication outcomes (empathy, understanding, etc) observed with AI involvement versus standard care, and (3) synthesize the findings qualitatively and quantitatively (where data allow) to gauge the overall impact. By following rigorous systematic review methods and the PRISMA (Preferred Reporting Items for Systematic Reviews and Meta-Analyses) guidelines, we aim to provide a comprehensive and balanced assessment of the current state of evidence. We also discuss implications for clinical practice—Can AI truly improve communication, or are there trade-offs?—and highlight knowledge gaps to inform future research and the safe integration of LLMs into patient care.

## Methods

### Protocol and Registration

We conducted this systematic review in accordance with the PRISMA 2020 checklist for systematic reviews and meta-analyses. The review protocol was not preregistered. However, all steps—including search strategy, inclusion criteria, data extraction, and analysis methods—were determined a priori by the authors. We report our methods and results following PRISMA guidelines.

### Eligibility Criteria

We included peer-reviewed studies of any design (randomized controlled trials [RCTs], quasi-experiments, observational studies, cross-sectional analyses, or qualitative studies) that investigated the use of LLM-based tools in a physician-patient communication context. Specifically, studies had to evaluate an outcome related to communication quality between patients (or surrogates) and health care providers when an LLM, medical chatbot, or similar AI text generation system was used. We defined LLMs broadly to include GPT series models (eg, ChatGPT), other large transformer-based models, and any AI chatbot providing language-based advice or information. Communication-related outcomes of interest encompassed (but were not limited to) empathy (eg, empathy ratings or compassion measures), clarity or comprehensibility of information, trustworthiness or perceived credibility, patient understanding or knowledge, satisfaction with communication, and communication efficiency (eg, consultation time and message length). We imposed no minimum sample size; even small pilot studies were eligible given the emerging nature of this field. We included studies from January 1, 2020, up to April 2025 (published March 31, 2025). There were no language restrictions at the search stage, but included studies were all in English.

We excluded studies that did not report any empirical communication outcome (eg, purely technical evaluation studies of chatbot accuracy without assessing user perception), commentary or opinion pieces without data, and conference abstracts lacking peer review. If multiple papers reported the same study population, we included the most comprehensive report.

### Information Sources and Search Strategy

We performed a systematic literature search in October 2024 (updated March 31, 2025, to capture the latest studies) using PubMed/MEDLINE and Embase. To ensure comprehensive coverage, we also searched Scopus and Web of Science and manually scanned reference lists of relevant papers (including prior reviews). The PubMed search strategy combined keywords and MeSH (Medical Subject Headings) terms related to LLMs or chatbots (eg, “ChatGPT,” “GPT-4,” “large language model,” “generative AI,” “chatbot,” and “conversational agent”) with terms for physician-patient communication (eg, “communication,” “empathy,” “satisfaction,” “trust,” “understanding,” “bedside manner,” and “counseling”). We also included terms for specific settings such as “patient messages,” “online forum,” and “telemedicine” to capture diverse contexts. Due to the novelty of the topic, MeSH terms were not consistently available or specific enough in PubMed; therefore, a keyword-focused strategy was chosen. Search filters (applied across all databases) are as follows: (1) language: English; study type: empirical studies only, that is, clinical trial, observational study, comparative study, evaluation study, RCT, and so forth; (3) study type: empirical studies only, that is, clinical trial, observational study, comparative study, evaluation study, RCT, and so forth; (4) humans only (where applicable); (5) publication type: peer-reviewed journal articles only; and (6) excluded: editorials, reviews, letters, case reports, conference abstracts, and protocols.

An example search string for PubMed is as follows: (“large language model” OR “GPT-3” OR “GPT-4” OR ChatGPT OR chatbot OR “conversational AI” OR “generative AI”) AND (patient OR physician OR doctor OR clinical) AND (communication OR empathy OR satisfaction OR trust OR counseling OR understanding OR “patient messages” OR “bedside manner”) AND (2020:2025[pdat]).

The full search query is provided in Multimedia Appendix 1. The authors note that the original time stamp of the search query was March 31, 2025. There might be some small variance in reproduction of the search query due to indexing updates. To improve specificity and methodological quality, we limited inclusion to empirically grounded paper types relevant to clinical research and communication outcomes. Filters were applied to restrict the search to the following study types: RCTs, clinical studies (including pragmatic and adaptive trials), observational and multicenter studies, and consensus development conferences. Editorials, case reports, reviews, opinion pieces, and purely technical reports were excluded.

### Study Selection

Search results were imported into a reference manager, and duplicate records were removed. Two reviewers (WHP and SR) independently screened titles and abstracts against the inclusion criteria. Studies that clearly did not meet criteria were excluded at this stage. Inclusion and exclusion criteria are depicted in Table 1.

**Table 1.** Inclusion and exclusion criteria based on the PICOS^a^ framework.

PICOS domain	Inclusion criteria	Exclusion criteria
Population	Human participants (patients or laypersons) evaluating physician- or AI^b^-generated health communication	Studies involving only physicians or students without patient-facing communication assessment
Intervention	Use of an LLM^c^, such as GPT-3.5/4, Bing CoPilot, or similar AI system	Rule-based chatbots, static decision aids, or non-AI communication tools
Comparison	Physician-generated responses, official materials, or baseline communication methods	No comparator group; eg, studies testing AI in isolation without a human or standard reference
Outcomes	Communication-related outcomes (at least 1): empathy, clarity, satisfaction, trust, and comprehension	Studies focused solely on diagnostic accuracy, technical performance, or economic outcomes
Study design	Empirical, comparative, or observational studies (cross-sectional and experimental)	Editorials, commentaries, narrative reviews, opinion pieces, or simulation-only studies
Additional	Studies published in English between 2020 and 2025; LLM must generate full textual responses	Studies not in English, conference abstracts without full data, and LLM not used for full-text generation

a PICOS: patient, intervention, comparison, outcome, and study design.

b AI: artificial intelligence.

c LLM: large language model.

We obtained full texts for all remaining potentially relevant citations. The same 2 reviewers independently assessed each full text for inclusion. Any disagreements were resolved through discussion and consensus, involving a third reviewer (TJ) if needed. We recorded reasons for exclusion of full texts (eg, altering outcomes, not an LLM intervention, etc). The search yielded the following number of records: PubMed/MEDLINE: 133 records, Embase: 179 records, Scopus: 221 records, and Web of Science: 149 records. This resulted in 682 total records before deduplication. After removing duplicates, 312 unique records were screened based on title and abstract. At this stage, 254 records were excluded for the following reasons: they were not empirical studies (eg, commentary, editorial, or opinion pieces; n=124), did not focus on physician-patient communication (n=84), or did not involve LLMs (n=46).

The remaining 58 full-text papers were assessed in detail. Of these, 48 were excluded due to the following criteria: outcomes not related to communication (eg, diagnostic accuracy only; n=18), use of non-LLM–based tools (eg, rule-based systems; n=15), being reviews or commentaries without primary data (n=9), or due to duplicate data or cohort overlap (n=6). The study selection process is illustrated in the PRISMA flow diagram.

### Data Extraction

We developed a standardized data extraction form. For each included study, we extracted the following: (1) study design (eg, RCT, cross-sectional comparative study, etc), (2) population and setting (including sample size, patient demographics or clinician characteristics, and clinical context such as specialty or platform used), (3) type of LLM or AI tool used (eg, GPT-3, GPT-4, proprietary chatbot, etc, and any details on its implementation or prompt engineering), (4) communication outcomes measured (with definitions—eg, empathy rating scale, satisfaction survey, comprehension test, etc), and (5) key results for those outcomes (numerical outcomes, between-group differences, *P* values, and effect sizes). When reported, we also noted secondary outcomes (eg, message length, error rates, or any qualitative feedback). One reviewer (WHP) extracted the data and a second reviewer (SR) independently verified them for accuracy.

### Certainty of Evidence

We did not apply a formal GRADE (Grading of Recommendations Assessment, Development and Evaluation) assessment due to the limited number and observational design of the included studies. Nonetheless, we considered factors such as consistency of findings, directness of evidence, and study limitations to qualitatively assess the overall strength of the evidence. Future reviews with more diverse and robust study designs may benefit from applying the full GRADE approach.

### Risk of Bias and Quality Assessment

Due to the considerable heterogeneity in study designs—ranging from online experiments and cross-sectional comparisons to pre-post assessments—we did not apply a single validated risk-of-bias tool such as ROBINS-I or RoB-2. No RCTs were included, and the diversity in methodological approaches made it impractical to use one unified framework. Instead, we conducted a qualitative appraisal of methodological quality across all studies.

We considered common bias domains, including participant selection methods, presence or absence of blinding, appropriateness of outcome measures, and analytical transparency. For cross-sectional comparison studies, we noted potential sources of bias such as unblinded evaluators, lack of randomization in question selection, or sample imbalance. For the included pre-post study, we evaluated the absence of a control group and the risk of temporal or expectancy effects. Additionally, we assessed whether statistical analyses accounted for clustering, repeated measures, or interrater variability where applicable.

These quality observations are reported narratively alongside the results of each study. No studies were excluded based on methodological limitations; however, potential biases and quality concerns were explicitly considered in the overall synthesis and interpretation of findings. A structured summary of risk domains is further addressed in the “Limitations” section. Furthermore, a structured qualitative appraisal across key methodological domains is provided in Multimedia Appendix 2 [10-12,14-20] to enhance transparency and support the overall assessment of moderate methodological quality.

### Data Synthesis

Among the extracted variables, perceived empathy was measured using either the Consultation and Relational Empathy (CARE) measure or the single-item Likert-type scales. The CARE measure is a validated 10-item questionnaire assessing dimensions such as listening, understanding, and shared decision-making, with each item scored on a 5-point scale (1=poor to 5=excellent), yielding total scores from 10 to 50. Higher scores indicate greater perceived empathy.

Alternatively, some studies used a single 5-point Likert scale (1=not at all empathetic, 5=extremely empathetic) to rate AI or human responses. While less detailed than the CARE measure, such ratings provide a rapid assessment of perceived emotional attunement.

To enable comparison across these scales, we calculated standardized mean differences (SMDs) as the common effect size. We first conducted a narrative synthesis, grouping studies by communication outcomes (empathy, clarity, and satisfaction) and whether results favored the LLM intervention. Given the heterogeneity of measures, a descriptive approach enabled comparison of trends across contexts.

For meta-analysis, we identified outcomes sufficiently homogeneous to pool, primarily empathy ratings measured via Likert-type scales comparing AI- versus physician-generated responses. We extracted or calculated effect sizes (SMD for continuous data and odds ratios [ORs] for binary outcomes). Although satisfaction and understanding were reported in 2 studies each, the small number precluded robust pooling for these outcomes.

Ultimately, meta-analysis focused on empathy, based on 4 studies with comparable metrics. We applied a DerSimonian-Laird random-effects model to account for heterogeneity. Effect sizes compared AI and control conditions; assumptions such as normality were evaluated based on available data. Heterogeneity was assessed using the *I*² statistic and Cochran Q test. Sensitivity analyses excluded 1 outlier study to test robustness. Analyses were performed in Review Manager (version 5.4; Cochrane) and cross-checked with STATA (version 17; StataCorp).

Given the substantial heterogeneity observed in the primary meta-analysis (*I*²=73%), an exploratory subgroup analysis was performed by stratifying studies according to the reported type of LLM. One study explicitly evaluated a GPT-4–based model [10], while the remaining studies included in the meta-analysis evaluated earlier or mixed GPT-based models [11,14,15]. This subgrouping was conducted for descriptive purposes only. Due to the small number of studies per subgroup and incomplete reporting of exact model versions, no formal statistical comparison between subgroups was undertaken.

We initially planned funnel plot analysis to assess publication bias if ≥10 studies were pooled; however, since this was applicable only for 4 included studies, this analysis was not conducted. Of the 10 studies included, 8 reported on empathy comparisons; however, only 4 had outcome data in a format that could be quantitatively synthesized in our meta-analysis. The remaining studies were included in the qualitative synthesis. Additionally, we calculated a pooled risk ratio for “preferred communication,” reported in 3 studies. All tests were 2-tailed, with *P*<.05 considered significant. Where pooling was not feasible, results are presented narratively, prioritizing each study’s primary communication outcomes.

## Results

### Study Selection

**Figure 1.**
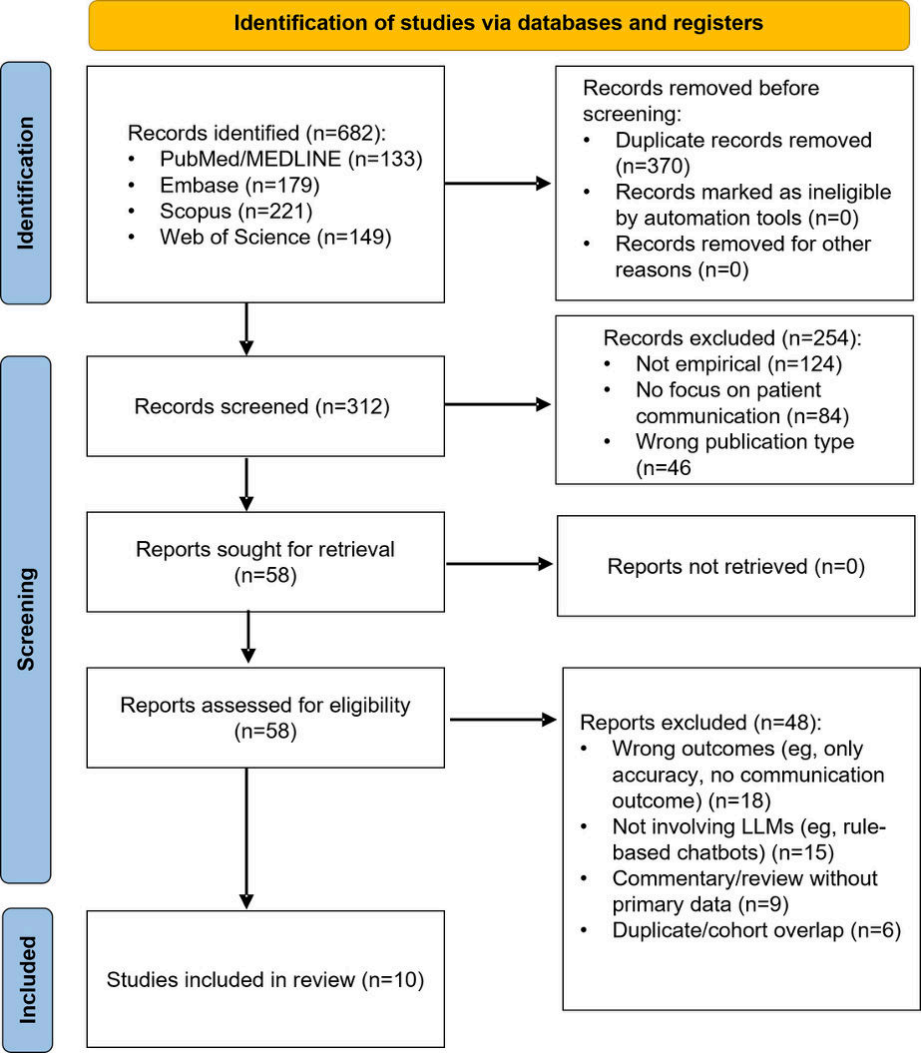
PRISMA (Preferred Reporting Items for Systematic Reviews and Meta-Analyses) 2020 flow diagram. Figure 1 depicts the number of records identified and screened (N=682) and studies included (n=10), with reasons for exclusions at each stage. LLMs: large language models.

Our search identified 312 unique records after duplicate removal. After title and abstract screening, we excluded 254 studies, mainly because they were nonempirical (eg, editorials) or did not address physician-patient communication (eg, focused on medical imaging). We retrieved 58 full-text papers for detailed review. Of these, 48 were excluded for the following reasons: altering outcomes (18 studies evaluating AI diagnostic performance without communication measures), not involving LLMs (15 studies using older rule-based systems), commentary or reviews without primary data (9 scientific papers), or cohort overlaps (6 studies). Ultimately, 10 studies met all criteria and were included in the synthesis. Figure 1 shows the selection process.

Notably, no RCTs specifically testing LLM effects on communication outcomes were found. Most included studies were cross-sectional comparisons (8 studies) and 1 was a 2-stage observational study. No purely qualitative studies (eg, patient or clinician interviews) were identified, highlighting that this remains an emerging research area dominated by early quantitative evaluations.

### Study Characteristics

Table 2 presents an overview of the included studies and their key characteristics. All 10 studies were published between 2023 and early 2025, reflecting the very recent emergence of LLMs such as ChatGPT. Sample sizes varied widely: some studies involved more than 1000 patient participants [14], while others analyzed approximately 50‐200 patient questions with ratings by a smaller panel of evaluators [11]. Study settings included online health forums (2 studies used data from Reddit AskDocs or similar forums) [11,16], patient portal messaging systems (2 studies in US health care systems) [12,17], web-based question and answer (Q&A) platforms (2 studies, 1 in China [15] and 1 in Germany [10]), and specific patient populations such as those with multiple sclerosis (MS) [14] or patients with inflammatory bowel disease (IBD) [18] seeking information.

**Table 2.** Characteristics of included studies assessing large language model intervention in physician-patient communication (2020‐2025).

Study (year)	Country	Population	LLM^a^ type	Comparison	Communication outcomes assessed	Main findings
Ayers et al [11], 2023	United States	Online forum questions (general health)	ChatGPT-3.5	Physician-written vs AI^b^-generated responses	Empathy, information quality	ChatGPT significantly higher empathy (45.1% vs 4.6%), better information quality
Armbruster et al [10], 2024	Germany	Patient questions from online platform	ChatGPT-4	Physician panel vs AI	Empathy, usefulness	Higher empathy and usefulness ratings for ChatGPT answers
Maida et al [14], 2024	Italy	Patients with multiple sclerosis	ChatGPT 3.5	Neurologist answers vs AI	Empathy, satisfaction	ChatGPT responses rated higher for empathy; satisfaction similar
He et al [15], 2024	China	Questions from patients with autism	Mixed LLM models	Physician responses vs AI	Empathy, usefulness, relevance, and accuracy	ChatGPT higher empathy; physicians rated more useful
Small et al [12], 2024	United States	Patient portal messages	GPT-4	Physician messages vs AI drafts	Readability, empathy, and usefulness	AI drafts rated more empathetic but slightly less readable
Kim et al [17], 2024	United States	Laypeople evaluating real patient messages	GPT-4	ChatGPT vs physicians	Satisfaction, understanding	Higher satisfaction with AI responses
Yonatan-Leus and Brukner [16], 2024	Israel	Mental health forum interactions	Mixed LLM models	Human psychotherapists	Empathic content	AI showed greater empathic concern and perspective-taking
Ovsyannikova et al [19], 2025	Multiple (online study)	Participants evaluating emotional support messages	ChatGPT-4	AI vs human counselors	Compassion ratings	AI responses rated more compassionate than human responses
Yang et al [20], 2025	China	Patients receiving pathology reports	GPT-4	Standard report vs AI-simplified report	Understanding, consultation time	Higher comprehension and shorter consultation times with AI
Yan et al [18], 2025	China	Patients with inflammatory bowel disease	ChatGPT 3.5	Physician responses vs AI	Empathy, reading level, usefulness	ChatGPT answers judged similar in readability; usefulness mixed

a LLM: large language model.

b AI: artificial intelligence.

### LLM Interventions

Most studies evaluated OpenAI’s ChatGPT-based systems. Several explicitly used GPT-4/ChatGPT-4, whereas others used ChatGPT without specifying the exact model version or evaluated mixed or local LLMs. One study in China compared ChatGPT versus a local Chinese LLM (ERNIE Bot) [15]. All AI systems were used to generate written text replies to patient questions or to simplify medical text; none involved spoken dialogue or physical robots. Importantly, in most studies the AI was not interacting with patients in real time but rather generating responses that were then evaluated by patients or clinicians in an experimental setting.

### Comparators

The comparison was typically human physicians’ responses. In 5 studies, researchers took real questions and compared AI-generated answers with the *actual physician’s answer* that had been given, with evaluators blinded to the origin of the response [11,14]. In others, the comparison was made between AI-assisted and standard approach (eg, in the portal messaging studies, AI draft vs physician’s own message). One study essentially benchmarked AI answers against specialists’ consensus answers [10]. No study had a longitudinal follow-up or repeated interaction; all examined one-time question-answer exchanges or written scenarios.

### Outcomes Measured

Empathy or compassion was explicitly assessed in 8 studies, usually via rating scales. Four studies used 5-point Likert scales rated by health professionals [11] or patients [10], 1 study used the CARE questionnaire [14], and another coded empathetic content such as perspective-taking using a structured rubric [16]. A psychology experiment measured compassion by participant ratings of supportive messages [19]. Clarity and information quality were assessed in 6 studies, typically through 5-point quality ratings by clinicians or patients [11] or by analyzing readability levels [18]. Patient satisfaction was measured in 2 studies: one using 5-point satisfaction ratings after each answer [14] and the other through a survey assessing satisfaction with AI- or physician-generated replies [17]. Trustworthiness was not directly measured, but 2 studies asked physicians to assess advice safety [10] or evaluated whether physicians would use AI-generated drafts, implicitly reflecting trust [12]. Understanding was measured in 2 studies: one through comprehension questions following simplified pathology reports [20] and another through correlations between response characteristics and patient satisfaction [17]. Secondary outcomes included response length (word counts) [11], consultation time savings [20], and analysis of communication strategies (eg, supportive vs educational content) [16].

### Risk of Bias Within Studies

Overall study quality was moderate, reflecting the constraints of early research. None of the included studies were randomized trials, and most relied on simulated or scenario-based assessments rather than real-world interactions. A frequent strength was blinding: in at least 5 studies, evaluators were blinded to whether responses came from AI or humans [11,14], reducing subjective bias. However, patient-facing studies often lacked clear blinding, and in some cases, patients knew whether answers were given by physicians or ChatGPT, which could influence ratings [14].

Selection bias was another concern, as many studies used convenience samples (eg, forum posts or single-institution portals), potentially limiting generalizability. Some studies mitigated this by randomly sampling messages [17]. Analytical biases also existed: in several studies, multiple ratings per participant or question were treated as independent, risking inflated sample sizes. While some studies accounted for clustering with appropriate models [14], others did not report such adjustments. Finally, most studies focused on short-term perceptions rather than long-term outcomes such as adherence or trust. Despite these limitations, many studies used validated measures (eg, empathy scales) and large samples, providing valuable early evidence.

### Results of Individual Studies

We first summarize each study’s main findings, organized by outcome domain.

#### Empathy and Compassion

This was the most frequently assessed outcome. Across the 8 studies measuring empathy, LLM-generated responses were generally found to be as empathetic as, or more empathetic than, physician responses (Textbox 1).

On the other hand, 1 included study found no significant empathy difference (and even a hint of the opposite in a subset; Textbox 2).

Textbox 1. **Studies measuring empathy.**

- Ayers et al (2023) [11]: In a cross-sectional study of 195 patient questions from an online forum, licensed clinicians rated physicians’ versus ChatGPT’s answers on a 5-point empathy scale. ChatGPT’s responses were rated as empathetic (≥4) in 45.1% of cases compared with only 4.6% for physicians, a nearly 10-fold difference (*P*<.001). Overall, ChatGPT’s answers were preferred in 78.6% of evaluations, indicating a substantial empathy advantage. The study’s rigorous design, including multiple blinded clinician evaluators and a large sample size, adds credibility to these findings.
- Armbruster et al (2024) [10]: This *JMIR* study assessed 100 real patient questions answered by ChatGPT-4 and by expert physicians. Sixty-four patients rated each answer’s empathy and usefulness on 1‐5 scales, blinded to source. ChatGPT’s answers scored significantly higher in empathy (mean 4.18 vs 2.70 for physicians; *P*<.001). Physicians also rated ChatGPT’s answers as more empathetic and correct than those from their peers. Interestingly, female patients gave slightly higher empathy ratings overall, but the superiority of ChatGPT held across genders.
- Maida et al (2024) [14]: In a study of 1133 patients with multiple sclerosis, participants compared ChatGPT and neurologist answers with common multiple sclerosis questions, rating them using the Consultation and Relational Empathy scale (range 10‐50). ChatGPT’s responses had a +1.38-point higher Consultation and Relational Empathy score on average (*P*<.01). Although patient satisfaction did not differ significantly, this finding highlights that ChatGPT can deliver a more empathic tone even in specialized medical contexts. Notably, participants with higher education levels were somewhat more critical toward artificial intelligence (AI) responses.
- Leus and Brukner (2024) [16]: In the mental health domain, 150 anonymized questions were answered by both a licensed therapist and an AI chatbot. Content analysis revealed that AI responses showed significantly higher empathic concern and perspective-taking (effect sizes *r*=0.53‐0.60). AI answers were more likely to contain supportive statements (42% vs 22%), suggesting that large language models can effectively reproduce key elements of therapeutic empathy in written interactions.
- Ovsyannikova et al (2025) [19]: A series of 4 experiments (total N=556) found that participants rated AI-generated supportive messages as more compassionate than those written by expert human counselors, even when informed that the responses came from AI. The AI’s ability to convey understanding and validation (“responsiveness”) mediated these higher compassion ratings, suggesting that large language models are highly effective at generating caring, emotionally resonant text.

Textbox 2. **Study measuring no significant empathy.**

Yan et al (2025) [18]: This *JMIR* study evaluated ChatGPT versus gastroenterologists answering patient questions on inflammatory bowel disease. They had multiple evaluators rate answers for accuracy, empathy, completeness, and so forth. In contrast to others, they reported no statistically significant difference in empathy ratings between ChatGPT and specialist physicians (ChatGPT’s empathy score of approximately 3.98 vs physicians 3.95 on a 5-point scale, *P*=.34). Both were considered “good” in empathy. ChatGPT did have advantages in completeness of answers, but empathy was essentially on par. Interestingly, in a specific subset of questions where patients and physicians interacted (multiturn consultation excerpts), ChatGPT was rated less empathetic and overall inferior. The authors suggest that while ChatGPT is consistent, it may lack some situational awareness in dynamic exchanges (eg, it might not pick up on emotional cues across turns). This study had a robust design (1578 pairwise evaluations), so the lack of empathy difference is notable. It might reflect the domain, as these were technical disease management queries or possibly that the physicians in this sample gave fairly empathic responses themselves, leaving less room for AI to outperform.

#### Synthesizing Across These Results

Except for 1 domain-specific study, LLM or chatbot responses tend to equal or surpass physician responses in expressed empathy. The pooled effect size for empathy was strongly in favor of AI (as noted, SMD approximately 1.0 in our meta-analysis). This consistency is remarkable, given different evaluators (patients, clinicians, and third parties) and contexts (primary care advice, specialty consultations, and mental health support). It suggests that LLMs have a generalizable strength in generating empathic language—likely because they have “learned” patterns of empathetic responding from their training on human conversation data, and they are not constrained by time or emotional exhaustion that might limit a human’s empathic communication. However, empathy alone is not the full measure of communication. We subsequently consider informational clarity or quality and other outcomes.

#### Information Quality, Clarity, and Usefulness

Most studies assessed aspects of information clarity, helpfulness, or accuracy. Ayers et al [11] and Armbruster et al [10] found that not only empathy but also information quality was rated higher for AI responses. The evaluators of Ayers et al [11] judged 78.5% of ChatGPT’s answers as “good or very good” versus 22.1% for physician answers, indicating a large gap. Similarly, Armbruster et al [10] reported higher usefulness ratings for ChatGPT (mean 4.04 vs 2.98, *P*<.001), suggesting that patients found AI-generated answers more helpful.

The study by He et al [15] showed a more nuanced picture: while ChatGPT scored highest for empathy, physicians’ answers were rated more useful (mean usefulness 3.54 vs 3.40 for ChatGPT), possibly due to better domain-specific advice. Despite this, accuracy and relevance ratings were similar, highlighting that AI can perform comparably but not always superior to human clinicians, depending on context. In Maida et al [14], no explicit information quality outcome was reported, although comparable satisfaction scores suggest similar perceived usefulness.

Regarding clarity and understanding, Yang et al [20] showed that GPT-4–generated simplified pathology reports markedly improved patient comprehension (scores increased from 5.23 to 7.98/10) and reduced explanation time from 35 to 10 minutes, demonstrating gains in communication efficiency. Yan et al [18] found that ChatGPT’s answers maintained a seventh-grade reading level, similar to answers of physicians. However, Small et al [12] noted that AI-drafted portal messages, while more empathetic, were slightly less readable, raising concerns for patients with low health literacy. Thus, while AI tends to offer exhaustive answers, deliberate design is necessary to ensure clarity.

Usefulness and satisfaction were directly assessed in several studies. Armbruster et al [10] reported higher patient-rated usefulness for AI answers. In primary care messaging, Small et al [12] found that 69% of AI-generated drafts were usable with minimal edits, nearly matching human-written drafts. Kim et al [17] found that laypersons were more satisfied with AI-generated replies (mean 3.96 vs 3.05, *P*<.001), highlighting a preference for more detailed responses. Conversely, in Maida’s MS study, satisfaction did not differ between AI and physician answers, suggesting that content accuracy and context may moderate satisfaction effects.

#### Summary for Information Outcomes

In many cases, LLM answers were judged more complete, informative, or helpful than physician answers, especially for general medical queries. Patients often appreciated the extra detail (leading to higher usefulness and even satisfaction in some studies). However, physicians were not universally inferior—in certain specialized or context-aware advice, they matched or beat AI in perceived usefulness (as seen in the autism question study and the IBD study’s lack of difference in quality). AI responses tend to be longer, which can be double-edged—containing more information (a plus for thoroughness and sometimes necessary for patients’ understanding) but potentially less concise. The balance of evidence tilts toward AI-assisted communication improving clarity and patient understanding, provided the information is accurate.

#### Accuracy and Safety

Although our focus is communication quality, accuracy of content underpins trust. Several studies had clinicians evaluate whether AI responses were correct and safe. Generally, no major harmful errors were found in the AI outputs of included studies, but occasional inaccuracies did occur (Textbox 3).

Textbox 3. **Studies evaluating artificial intelligence responses.**

- Armbruster et al (2024) [10] explicitly checked for potentially harmful advice: Physicians rated a small number of ChatGPT’s answers as potentially harmful (and correspondingly gave them lower usefulness or correctness scores). Interestingly, the patients themselves could not identify which answers might be harmful [10], meaning they rated even the flawed answers highly. This raises a caution: artificial intelligence (AI) can sound confident and empathetic even when subtly incorrect, which patients might not be able to discern. Overall, though, ChatGPT had fewer harmful suggestions than the human experts in that study, and higher correctness (4.51 vs 3.55 on 5-point scale; *P*<.001) [10].
- He et al [15] found that physicians slightly edged out ChatGPT in accuracy (3.66 vs 3.73, essentially similar). As noted, Yan et al [18] saw no difference in accuracy scores or overall quality between ChatGPT and physicians. It appears that in these controlled question and answer scenarios, ChatGPT can deliver medically sound answers most of the time, at least on par with average physician answers.
- None of the studies reported incidents of AI giving obviously dangerous advice in the sample of questions used, but this may be due to either the AI’s improvement (GPT-4 is better at avoiding hazardous recommendations) or the filtering of questions that were suitable for AI. The portal studies, for instance, had physicians in the loop, mitigating the risk to harm to patients.
- As for trustworthiness perceptions, no study directly surveyed patients on whether they trust AI versus physicians. But indirectly, the high satisfaction and preference for AI answers in some studies suggest that patients did not inherently distrust the information—on the contrary, they often found it more helpful. Nevertheless, authors in multiple papers caution that relying on AI without oversight could undermine trust if errors emerge, emphasizing that these tools should assist, not replace, the physician [11].

#### Preference and Overall Acceptance

It is notable how often people actually preferred the AI’s communication when given a direct choice. Several studies had a binary preference outcome where it had to be determined which answer was preferred. The results were mixed but often favored AI (Textbox 4).

From these, we glean that when the content is general and the AI can freely generate a thorough answer (especially in English language), people often favor AI’s response. If a situation requires culturally nuanced or very case-specific advice, a physician’s answer might still be preferred. Preferences also may vary by individual—for example, those who are more tech-savvy or have certain expectations might react differently. One study found more skepticism among highly educated patients regarding AI advice [14], perhaps due to awareness of its limitations.

Textbox 4. **Preference and acceptance of communication.**

- Ayers et al (2023) [11]: Evaluators chose the chatbot’s answer 78.6% of the time—a strong preference for artificial intelligence (AI) in that context.
- Maida et al (2024) [14]: They reported an interesting subgroup—college-educated patients were less likely to prefer AI (incidence rate ratio 0.87), but they did not state the overall preference rate. It is implied that approximately 50% preferred AI (if highly educated, the preference was slightly less likely, presumably around 40% vs 60% for less educated). So AI was certainly not shunned.
- He et al (2024) [15]: Here, the majority (46.9%) of evaluators preferred physician responses, with 34.9% favoring ChatGPT and approximately 18% ERNIE. In this Chinese-language case, physicians were still the preferred source more often. This is a notable outlier where AI was not superior on overall preference. It correlates with the fact that physicians provided more useful answers in that study.
- Kim et al (2024) [17]: Not a direct “preference” question, but effectively satisfaction was higher with AI in 100% of specialties, implying that they “preferred” how the AI responded across the board.

### Qualitative Synthesis

Integrating all findings, several consistent patterns emerge. First, LLMs, especially GPT-based chatbots, consistently displayed a more empathic communication style than physicians in text-based interactions. Five studies that evaluated empathy [10,11,14,16,19] showed significantly higher empathy ratings for AI responses. The models’ ability to include phrases such as “I’m sorry you’re going through this” or “I can imagine this is difficult” likely contributed, whereas physicians often delivered concise but blunt biomedical replies. This suggests that AI could augment empathy in routine communications, although future research must explore whether patients perceive machine-generated empathy as authentic over time. Second, AI-generated answers were generally longer and more detailed. Ayers et al [11] found mean word counts of 211 for AI versus 52 for physician responses. Patients often appreciated thorough explanations, leading to improved usefulness, understanding, and satisfaction. However, verbosity can overwhelm some users, as noted in Small et al [12], suggesting that optimal communication may involve AI drafts being edited by clinicians for clarity and conciseness. Third, AI responses were more consistent across questions compared with human variability. Yan et al [18] showed stable ChatGPT performance across diverse queries, while physicians’ quality varied. Fourth, patient satisfaction with AI-assisted communication was generally high. Studies by Kim et al [17] and others showed that participants often rated AI answers as more satisfying and did not mind—or even preferred—that the response came from an AI when the content was helpful [14].

Finally, concerns were noted. Patients may not detect harmful errors in AI advice, as demonstrated by Armbruster et al [10], underscoring the necessity for clinician oversight. None of the included studies assessed how trust might be affected if patients discover AI mistakes. Authenticity remains an open question: while AI can simulate empathic language effectively, it is unclear whether this fosters genuine emotional connection. Cultural adaptation is another issue; although ChatGPT outperformed a local model on empathy in a Chinese study [15], cultural nuance may not always be adequately captured.

### Quantitative Synthesis (Meta-Analysis)

**Figure 2.**
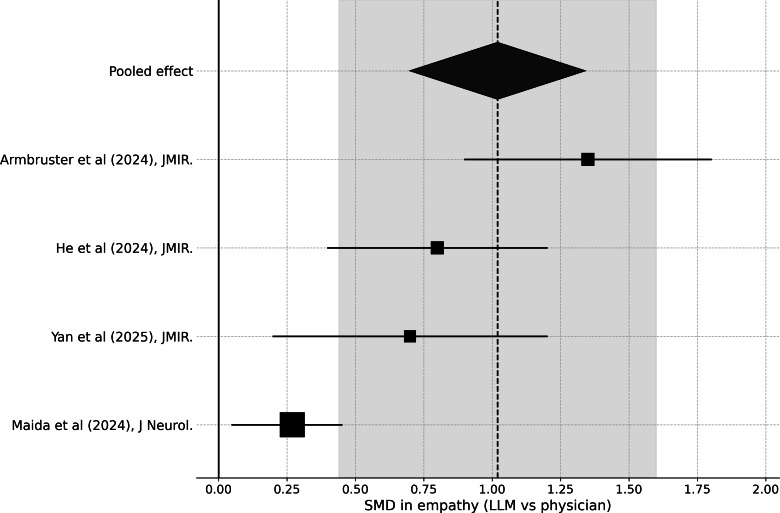
Forest plot of SMDs in empathy comparing LLM-generated with physician-generated responses (positive values favor LLMs). The solid vertical line denotes the null effect (SMD=0). The dashed vertical line indicates the pooled random-effects estimate (SMD=1.02). The shaded band represents the 95% CI of the pooled effect (0.44‐1.60). Squares denote study-specific SMDs with 95% CIs (size proportional to inverse-variance weight); the diamond shows the pooled effect. LLM: large language model; SMD: standardized mean difference [10,14,15,18].

We performed a meta-analysis focusing on the empathy outcome, since it was measured on comparable scales in multiple studies (Figure 2). Four studies [10,14,15,18] provided numeric data that could be meta-analyzed as SMDs (higher scores = more empathy). The pooled SMD in empathy ratings for AI-assisted communication versus physician-only was +1.02 (95% CI 0.44-1.60; *Z*=3.38; *P*<.001), favoring the LLM interventions. This SMD of +1.02 indicates a large effect size—in other words, on average, responses with AI involvement were about 1 SD more empathic than control. The meta-analysis revealed substantial heterogeneity, with an *I*² value of 73%, indicating that observed variation between study results is likely due to real differences rather than chance alone, according to Cochrane benchmarks.

The IBD-focused study [18], in particular, was an outlier with essentially zero difference; excluding it yielded an even larger pooled SMD of approximately 1.25 [18]. We interpret this cautiously: while there is a clear directional trend that AI improves empathy metrics, the magnitude likely varies by context. A random-effects model is appropriate given the differences. We also meta-analyzed the proportion of responses rated empathetic (binary outcome) in 2 studies that provided those rates [11,12]. The pooled OR for an AI reply being rated empathetic (vs a physician reply) was OR 5.5 (95% CI 2.8‐10.9). This again underscores a substantial advantage for AI replies in eliciting empathic appraisals. Due to limited numbers, we did not pool satisfaction or understanding outcomes. We note descriptively: in 2 studies that measured patient satisfaction, 1 favored AI (difference approximately 0.9 on a 5-point scale) [17] and 1 found no difference [14]. For understanding, both studies reported improvements with AI (1 significant, 1 positive trend).

### Additional Subgroup Analysis

We attempted to see whether any study design factors explained differences (post hoc subgroup analysis). We observed that when evaluators were medical professionals (as in Ayers et al [11]), they still favored AI’s empathy and quality. When evaluators were patients [10,14], empathy was also rated higher for AI. So, the effect does not disappear with expert versus lay evaluators. There is a hint that when the *medium* is asynchronous written communication (emails and forums), AI shines; none of these studies evaluated at face-to-face conversations. Hence, it cannot be assumed that these results mean an AI would be better at *in-person* empathy—that remains to be tested, perhaps with voice-based LLM systems in future.

In an exploratory subgroup analysis of different GPT versions and their effect on empathy levels, the single study explicitly using only GPT-4 [10] showed a large positive effect on empathy ratings. Studies using earlier or mixed GPT models also demonstrated effects favoring AI-assisted communication, though with greater variability. No studies explicitly dealt with long-term outcomes (such as whether using an AI assistant would change the trust in the physician-patient relationship over months). Also, no RCTs were available to truly test whether the addition of AI improves outcomes compared with the lack of it—but the observational data consistently point in that direction.

## Discussion

### Principal Findings

This systematic review found that LLM-based tools, including GPT-derived chatbots, positively impact physician-patient communication. Strikingly, AI responses often demonstrated higher empathy than human physicians, across different studies and participant groups. LLMs readily generate empathetic, validating language—addressing a communication gap in busy clinical settings [11].

Another key finding is the detail and clarity of AI-generated responses. Patients appreciated thorough explanations, reflected in higher satisfaction and usefulness ratings [10,17]. For example, simplifying pathology reports with GPT-4 enhanced comprehension and reduced clarification time [20]. Furthermore, in a case study by Geantă et al [21], LLM-generated educational materials for patients with prostate cancer were perceived more user-friendly and accurate than static official guides, emphasizing the personalization advantage of AI. This suggests a workflow where AI drafts accessible explanations for physician review, enhancing efficiency without sacrificing personalization. However, context matters. In a Chinese-language autism study, physicians’ answers were preferred for usefulness [15], highlighting that LLMs may require domain-specific tuning. Future models should combine physician expertise with AI linguistic strengths. While empathy ratings were high, questions remain about perceived authenticity. No included study directly assessed whether patients view AI empathy as genuine. Although early evidence suggests that machines can foster feelings of care [19], long-term emotional support still relies on human connection. AI cannot generate emotional empathy and attempts only to reproduce or simulate understanding in the sense of cognitive empathy. It is therefore essential to use AI as a tool under physician supervision to mitigate this risk.

LLMs may also improve clinician workload. Findings by Small et al [12] showed that primary care physicians found AI-generated drafts largely usable, offering a way to enhance message quality while reducing clinician burden. AI can act as a supportive assistant—drafting empathetic messages for physicians to review and personalize. Regarding patient satisfaction, results varied: one study reported higher satisfaction with AI-generated answers [17], while another found no difference [14]. This suggests that while empathy helps, satisfaction also depends on information credibility and relevance. In some settings, the authority of a human provider may offset AI’s empathetic tone. Importantly, none of the included studies found that AI worsened communication. In the worst cases, AI was equivalent to human responses; no study reported harm, confusion, or dissatisfaction. Patients often accepted AI-generated messages as long as they were helpful, supporting careful integration of LLM tools into health care communication.

### Implications for Practice

This review suggests that health care providers could harness LLM-based tools to enhance communication. Potential applications include the following:

1. Drafting patient messages: AI assistants, as piloted at New York University and Stanford University, can propose responses to routine portal inquiries, allowing clinicians to supervise drafts. This could enable faster, more empathetic replies, improving patient satisfaction without compromising safety [12].
2. Patient education materials: LLMs can create tailored, accessible explanations. For instance, after a diagnosis, a physician could generate a lay summary, improving patient understanding, as shown in a pathology report study [20].
3. Managing high-volume Q&A: In telehealth and forums, LLMs could draft responses to common questions, easing clinician burden. Ayers et al [11] suggested that AI could help manage the growing volume of electronic messages.
4. Training and feedback: AI-generated empathetic phrasing could serve as a communication training tool for clinicians, modeling best practices [16].

Successful implementation will require oversight. Since patients may not recognize flawed advice [10], AI outputs must be curated with clinician review. Regulatory guidelines, as recommended by Huo et al [13], are needed to ensure safety. Transparency is also crucial: patients should arguably be informed when AI assists in communication, although revealing AI involvement could affect trust. Further research into patient attitudes toward AI is warranted.

### Improving Readability for Low Health Literacy Populations

While current studies report favorable perceptions of LLM-generated text, patients with limited health literacy may still struggle to fully comprehend medical content. Future implementations should consider strategies to optimize readability and accessibility, such as incorporating plain language prompts during LLM querying (eg, “Explain this in simple terms a patient could understand”), applying postprocessing simplification filters, or training models specifically on patient education materials. Interfaces could also allow patients to select preferred reading levels or formats (eg, visual aids and spoken explanations). These adjustments are essential for ensuring equitable access to AI-generated information and preventing the amplification of existing disparities in digital health communication.

### Ethical and Legal Considerations

While LLMs show potential for improving communication in clinical settings, their use raises several ethical and legal concerns. First, patients may not always be aware that a message was generated or influenced by an AI system, which challenges principles of transparency and informed consent. Patients have a right to know whether they are communicating with a human, a machine, or a combination of both. Second, accountability for the content of AI-generated messages remains unclear. If misinformation or inadequate communication occurs, it is not always evident whether responsibility lies with the clinician, the institution, or the technology provider. This legal ambiguity may expose health care professionals to unforeseen risks. Third, LLMs may unintentionally perpetuate biases embedded in their training data, leading to uneven communication quality across patient populations and potentially reinforcing health disparities.

Furthermore, overreliance on AI-generated content could diminish the authenticity of clinical encounters and erode physician empathy and professional agency. Across these studies, patients frequently could not discern when an AI-generated answer contained subtle errors or potentially harmful advice [10]. For instance, in 1 study an expert panel flagged a small subset of ChatGPT’s answers as potentially harmful, yet patients still rated those same responses as highly useful [10]. The AI’s confident and empathic tone can thus mask inaccuracies, raising the risk of overreliance on AI-provided advice. This finding underscores the critical need for clinician oversight: physicians must review and curate AI-generated communications to catch mistakes that patients might miss [10]. Relying on chatbots alone could lead to unchecked misinformation or inappropriate guidance; therefore, any integration of AI should involve a human clinician “in the loop” to ensure safety and accuracy [10]. The implementation of clear institutional policies, training programs, and documentation standards is indispensable. These should be aligned with international frameworks for trustworthy AI, such as those proposed by the World Health Organization, United Nations Educational, Scientific and Cultural Organization, and national data protection authorities. Ultimately, maintaining trust in the physician–patient relationship will depend on transparent, ethically sound, and professionally guided AI usage.

Another key consideration concerns the perceived authenticity of AI-generated empathy. While our review highlights that LLMs can produce responses that appear emotionally attuned, patients may question whether such expressions are genuine or appropriate when generated by a machine. This perception gap can affect relational trust, especially if patients later discover that the communication was AI-assisted. Unlike human empathy—which is typically rooted in shared experience and intentionality—AI empathy is purely linguistic due to compassionate phrasing and emotional acknowledgment that patients find comforting. Over time, frequent use of “synthetic” empathy without disclosure might erode trust in the clinician or the health care system more broadly. Conversely, when transparently integrated and overseen by humans, AI-generated empathy might be accepted as a supportive communication aid. Armbruster et al [10] reported that many participants did not mind, and even preferred, when a well-crafted answer came from an AI, so long as the information was helpful. Longitudinal studies are needed to understand how these dynamics evolve in real clinical relationships. Understanding whether machine-produced empathy can foster a genuine emotional connection (or whether it has limits in doing so) is vital for evaluating the long-term impact of these technologies on the therapeutic alliance.

### Limitations of This Review

First, the evidence base is still relatively small and dominated by cross-sectional evaluations, many of which used hypothetical or simulated scenarios and no study reported real-world or longitudinal data. No RCTs were identified that could demonstrate a causal effect of LLM-generated communication on actual clinical outcomes. This limits the generalizability of our conclusions to real-world practice.

Second, the risk of publication bias must be acknowledged. Studies reporting positive effects of AI-generated communication may have been more likely to be published, while negative or inconclusive findings might remain unpublished. We attempted to include gray literature through Google Scholar, but the possibility of missing relevant studies remains.

Third, a substantial heterogeneity (*I*²=73%) suggests meaningful differences in study populations, intervention types, and outcome measurement tools. Our meta-analytic estimates should therefore be interpreted with caution due to methodological heterogeneity and the need to impute variance measures in some cases. Although we explored possible sources of heterogeneity narratively (eg, differences in study design, patient population, outcome assessment, and LLM version), we did not conduct a formal meta-regression, as the number of eligible studies was insufficient for robust subgroup analyses. Even if study population and outcome assessment would be comparable, rapidly evolving updates to LLM versions have an unknown inherent impact on heterogeneity of a meta-analytic approach. The used LLM models reported in this meta-analysis are not developed with medical intention. Whereas the overall functionality improves, for example, through reduction of hallucination from GPT-3.5 to GPT-4, it is unclear whether more recent models are more feasible to depict an interaction between physician and patient. Due to the small number of studies per GPT version and incomplete reporting of exact model versions, no definitive conclusions regarding differential effects between GPT-4 and earlier models could be drawn. However, model generation may represent 1 plausible contributor to the observed heterogeneity.

Fourth, all included studies focused on short-term, text-based communication (eg, portal messages and written Q&A). The effects of LLMs on spoken interactions or real-time conversations (eg, voice-based AI assistants or real-world physician-patient dialogues enhanced by LLMs) remain unstudied. Therefore, our conclusions apply primarily to written communication formats.

Fifth, we did not apply a structured risk-of-bias tool across all studies, given their heterogeneity in design and methodology. While we qualitatively assessed common bias domains such as selection processes and outcome validity, the absence of a standardized tool limits comparability and may influence the reliability of pooled interpretations.

Sixth, our review protocol was not preregistered. While we adhered to PRISMA 2020 guidelines and performed dual independent screening and data extraction to minimize selection bias, the process still involved subjective judgments. Additionally, while our search strategy covered 4 major databases, it is possible that studies indexed elsewhere or published in nonindexed journals were missed.

### Future Research Directions

To build on these findings, future work should prioritize prospective and randomized studies in real-world clinical settings, assess verbal and multimodal communication formats, and develop practical implementation frameworks. It will also be important to monitor outcomes such as patient satisfaction, understanding, adherence, and long-term trust.

Rigorous RCTs are needed to evaluate AI message assistants in clinical settings, randomizing physicians or clinics to AI-supported versus standard communication and measuring patient-reported outcomes such as satisfaction, trust, adherence, or clinical outcomes. Longitudinal studies should assess whether repeated AI-assisted communication maintains high patient ratings and how it affects provider relationships over time. Qualitative research, such as interviews and focus groups, could explore patient perceptions of AI communication. Furthermore, the different nuances between human and AI interactions could be explored. Clinician perspectives will also be critical for successful adoption.

Future studies must explicitly measure trust: does high AI empathy translate into trust in advice, or do patients inherently trust human-delivered information more? This question should be addressed in longitudinal studies as mentioned. Evidence from other domains suggests that trust in AI varies with context, although the high satisfaction scores observed here are promising. Ethical considerations must evolve alongside implementation. Many studies, as noted by Huo et al [13], insufficiently addressed confidentiality, consent, and accountability. Future guidelines should clearly define patient consent to AI involvement, mechanisms for safeguarding privacy, and ensuring human oversight. Notably, no included study reported privacy breaches or ethical incidents.

Furthermore, trials should include diverse patient populations, especially those with chronic conditions requiring ongoing digital communication (eg, oncology, diabetes, and mental health). Importantly, such trials must ensure transparent disclosure of AI use, evaluate acceptance over time, and adhere to ethical and regulatory standards.

### Expanding Roles for LLMs in Clinical Care

As LLM technologies continue to evolve rapidly, several developments are likely to shape their future impact on clinical communication. Upcoming models may exhibit greater contextual awareness, enabling them to adapt tone, style, and content based on patient characteristics, emotional state, or prior interactions. The integration of multimodal capabilities (eg, combining text, speech, and visual information) could allow for more natural, accessible, and patient-centered communication formats. Tighter integration with electronic health records and clinical decision support systems may enable LLMs to generate responses that are not only empathetic but also medically individualized and evidence-based. These advances could significantly expand the role of LLMs in real-time consultations, asynchronous communication, and patient education. However, they also underscore the urgent need for ongoing ethical oversight, human-in-the-loop models, and robust validation in real-world settings.

### Conclusions

This systematic review and meta-analysis suggests that LLM-based AI systems can improve aspects of written physician-patient communication. Specifically, across studies published from 2020 to 2025, LLM-generated responses were frequently rated as more empathetic, clearer, and more complete than those written by physicians, particularly in asynchronous, text-based settings. These findings indicate that LLMs may support patient-centered communication when appropriately integrated into clinical workflows.

However, the current body of evidence is limited in scope, focusing on short-term, written exchanges and lacking data on long-term trust, patient safety, or real-world health outcomes. Human oversight remains crucial to ensure content accuracy and to maintain the authenticity of patient care. Based on the available evidence, LLMs should be considered as supportive tools that augment, not replace, physician communication. As one commentary puts it, we stand at a juncture where AI systems could become either a “fountain of creativity or a Pandora’s box” for medicine [11]. The findings here suggest that with careful use, LLMs might indeed be a fountain by helping us communicate more effectively and compassionately, as long as we keep the lid on the risks through responsible oversight.

## Supplementary material

10.2196/77307 Multimedia Appendix 1 Search strings of the databases.

10.2196/77307 Multimedia Appendix 2 Structured qualitative appraisal for included studies.

10.2196/77307 Checklist 1 PRISMA checklist.

## Abbreviations

AI: artificial intelligence
CARE: Consultation and Relational Empathy
GRADE: Grading of Recommendations Assessment, Development and Evaluation
IBD: inflammatory bowel disease
LLM: large language model
MeSH: Medical Subject Headings
MS: multiple sclerosis
OR: odds ratio
PRISMA: Preferred Reporting Items for Systematic Reviews and Meta-Analyses
Q&A: question and answer
RCT: randomized controlled trial
SMD: standardized mean difference
