# Supplementary material for: Impact of Large Language Model–Based AI Tools on Physician-Patient Communication: Systematic Review and Meta-Analysis
Source: J Med Internet Res. 2026 Jul 31;28:e77307. doi: 10.2196/77307 (PMC13427064; doi:10.2196/77307)
Supplement: Multimedia Appendix 1 [file jmir-v28-e77307-s001.docx]

## Multimedia Appendix 1. Search strings of the databases.

The full queries used for this study are provided below. Data was retrieved from January First 2020 until March 31^st^ 2025. It is noted by the authors, that slight discrepancies in reproduction of the query might be caused through routine indexing updates.

| **Database** | **Search Strategy (January 2020 – March 2025)** |
| --- | --- |
| **PubMed/MEDLINE** | (    ("large language model"[tiab] OR "large-language model"[tiab] OR LLM[tiab] OR     "Generative Pre-trained Transformer"[tiab] OR ChatGPT[tiab] OR "GPT-3"[tiab] OR "GPT-4"[tiab])    AND    (patient*[tiab] OR physician*[tiab] OR doctor*[tiab] OR clinician*[tiab])    AND    (communication OR communicat*[tiab] OR empath*[tiab] OR satisf*[tiab] OR trust*[tiab] OR counsel*[tiab] OR understand*[tiab]       OR ("patient"[tiab] AND (message*[tiab] OR portal[tiab] OR inbox[tiab] OR "in-basket"[tiab]))       OR (online[tiab] AND forum*[tiab]) OR telemedicine[tiab] OR "bedside manner"[tiab])  )  AND ("2020/01/01"[Date - Publication] : "2025/03/31"[Date - Publication])  AND english[lang]  AND (    "Clinical Study"[pt] OR    "Clinical Trial"[pt] OR    "Clinical Trial, Phase II"[pt] OR    "Clinical Trial, Phase III"[pt] OR    "Clinical Trial, Phase IV"[pt] OR    "Controlled Clinical Trial"[pt] OR    "Randomized Controlled Trial"[pt] OR    "Observational Study"[pt] OR    "Multicenter Study"[pt] OR    "Pragmatic Clinical Trial"[pt] OR    "Comparative Study"[pt] OR    "Evaluation Study"[pt]  ) |
| **EMBASE** | Interface & database. Embase (Ovid). MEDLINE records were not excluded.  Search date. 31 March 2025.  Date limit. Date Created (dc) from 01 Jan 2020 to 31 March 2025.  Fields. .ti,ab,kf. (title, abstract, keyword heading).  Subject mapping. Map Term to Subject Heading off.  Publication types. No positive selection; we excluded editorials, letters, notes, and conference abstracts.  Stepwise Ovid syntax (enter each line separately):   1. (chatgpt or "gpt-3*" or "gpt-4*" or "large language model*").ti,ab,kf. 2. (patient* or physician* or doctor* or clinician*).ti,ab,kf. 3. ((communication* or empath* or satisfaction or trust* or "shared decision*") adj5 (patient* or physician* or doctor* or clinician* or message* or portal* or forum*)).ti,ab,kf. 4. 1 and 2 and 3 5. limit 4 to dc=20200101-20250331 6. limit 5 to english language 7. limit 6 to human 8. 7 not (editorial or letter or note or "conference abstract").pt.   Yield at time of search (31 March 2025):  Step 1: 16,067; Step 2: 14,430,256; Step 3: 228,680; Step 4: 543; Step 5: 269; Step 6: 263; Step 7: 258; Step 8 (final set): 170. |
| **Scopus** | TITLE-ABS-KEY((chatgpt OR "gpt-3" OR "gpt-4" OR "large language model*") AND (patient* OR physician* OR doctor* OR clinician*) AND ((communication* OR empath* OR satisfaction OR trust* OR "shared decision*") W/5 (patient* OR physician* OR doctor* OR clinician* OR message* OR portal* OR forum*))) AND PUBYEAR > 2019 AND PUBYEAR < 2026 AND (LIMIT-TO(SRCTYPE,"j")) AND (LIMIT-TO(DOCTYPE,"ar") OR LIMIT-TO(DOCTYPE,"ip")) AND (LIMIT-TO(LANGUAGE,"English")) AND (LIMIT-TO(SUBJAREA,"MEDI")) AND (LIMIT-TO(EXACTKEYWORD,"Human")) AND (LIMIT-TO(PUBSTAGE,"final")) |
| **Web of Science** | **Interface & indexes.** Web of Science **Core Collection**; indexes included: **SCI-EXPANDED, SSCI, ESCI** (conference/book indexes not selected). **Search date.** 31 March 2025. **Fields.** **Topic** (TS=) — title, abstract, author keywords, Keywords Plus. **Language / document type.** **English**; **Article** only (Early Access excluded). **Day-level date cut-off.** Publication date DOP=2020-01-01/2025-03-31.  **Advanced Search query (single line):**  TS=((chatgpt OR "gpt-4*") AND (patient* OR physician* OR doctor* OR clinician*) AND ((communication* OR empath* OR satisfaction OR trust* OR "shared decision*") NEAR/2 (patient* OR physician* OR doctor* OR clinician*))) AND LA=(English) AND DT=(Article) AND DOP=2020-01-01/2025-03-31 |
