# Supplementary material for: Impact of Large Language Model–Based AI Tools on Physician-Patient Communication: Systematic Review and Meta-Analysis
Source: J Med Internet Res. 2026 Jul 31;28:e77307. doi: 10.2196/77307 (PMC13427064; doi:10.2196/77307)
Supplement: Multimedia Appendix 2 [file jmir-v28-e77307-s002.docx]

## Multimedia Appendix 2. Structured qualitative appraisal for included studies.

| \| **Study (Year)** \| \| --- \|  \|  \| \| --- \| | **Participant selection** | **Blinding status** | **Outcome measures** | **Analytical transparency** |
| --- | --- | --- | --- | --- | --- | --- |
| Ayers et al [11], 2023 | Patient questions sampled from a public online forum; evaluators were licensed clinicians. Sampling strategy and inclusion criteria described. | Evaluators were blinded to the origin of responses (AI vs physician). | Empathy and information quality rated using Likert-type scales. | Statistical methods described; comparative analyses reported. |
| Armbruster et al [10], 2024 | Patient participants recruited to evaluate AI-generated responses; recruitment method described. | No blinding reported (participants evaluated AI responses only). | Patient-rated empathy and usefulness using Likert-type scales; physician safety assessment reported. | Descriptive and comparative analyses reported. |
| Maida et al [14], 2024 | Patients with multiple sclerosis recruited from clinical settings; inclusion criteria reported. | Patients evaluated paired responses without disclosure of source. | Empathy assessed using CARE measure; satisfaction ratings reported. | Statistical analyses including subgroup analyses described. |
| He et al [15], 2024 | Web-based patient questions included; selection process described. | Blinding of evaluators not explicitly reported. | Empathy, usefulness, relevance, and accuracy rated using numeric scales. | Comparative statistical analyses reported. |
| Small et al [12], 2024 | Real patient portal messages from a single health system included; inclusion process described. | No blinding reported (physicians aware of AI drafts). | Readability metrics and clinician-rated empathy/usefulness reported. | Analytical approach and outcome reporting described. |
| Kim et al [17], 2024 | Lay participants recruited to evaluate patient–physician messages; recruitment method reported. | Blinding to response source not consistently reported. | Satisfaction and perceived understanding rated on Likert scales. | Statistical comparisons across specialties reported. |
| Yonatan-Leus and Brukner [16], 2024 | Online mental health support transcripts selected; selection criteria described. | Blinding of coders to response source reported. | Empathic content assessed via structured content analysis. | Coding framework and analytical approach reported. |
| Ovsyannikova et al [19], 2025 | Online participants recruited to rate supportive messages; recruitment method described. | Evaluators blinded to AI vs human origin. | Compassion ratings using numeric scales reported. | Statistical analyses and mediation analyses reported. |
| Yang et al [20], 2025 | Patients receiving pathology reports recruited; inclusion criteria reported. | No blinding reported (intervention vs standard report). | Objective comprehension scores and consultation time measured. | Comparative analyses between groups reported. |
| Yan et al [18], 2025 | Patient education materials evaluated; selection process described. | Blinding of evaluators to response source reported. | Readability indices and usefulness ratings reported. | Statistical comparison methods described. |
